# Advances in the Pathogenesis of Alzheimer’s Disease: Focusing on Tau-Mediated Neurodegeneration

**DOI:** 10.1186/2047-9158-1-24

**Published:** 2012-12-15

**Authors:** Yale Duan, Suzhen Dong, Feng Gu, Yinghe Hu, Zheng Zhao

**Affiliations:** 1 Key Laboratory of Brain Functional Genomics, Ministry of Education,Shanghai Key Laboratory of Brain Functional Genomics, East China Normal University, 3663 Zhongshan Road (N), Shanghai 200062, China; 2 Shanghai Engineering Research Center for Molecular Therapeutics and New Drug Development, East China Normal University, Shanghai 200062, China

**Keywords:** Alzheimer’s disease, Tau, A-beta, Tauopathy, Tau hyperphosphorylation, Intraneuronal neurofibrillary tangles

## Abstract

In addition to senile plaques and cerebral amyloid angiopathy, the hyperphosphorylation of tau protein and formation of intraneuronal neurofibrillary tangles (NFTs) represents another neuropathological hallmark in AD brain. Tau is a microtubule-associated protein and localizes predominantly in the axons of neurons with the primary function in maintaining microtubules stability. When the balance between tau phosphorylation and dephosphorylation is changed in favor of the former, tau is hyperphosphorylated and the level of the free tau fractions elevated. The hyperphosphorylation of tau protein and formation of NFTs represent a characteristic neuropathological feature in AD brain. We have discussed the role of Aβ in AD in our previous review, this review focused on the recent advances in tau-mediated AD pathology, mainly including tau hyperphosphorylation, propagation of tau pathology and the relationship between tau and Aβ.

## Review

### Introduction

**Figure 1:**
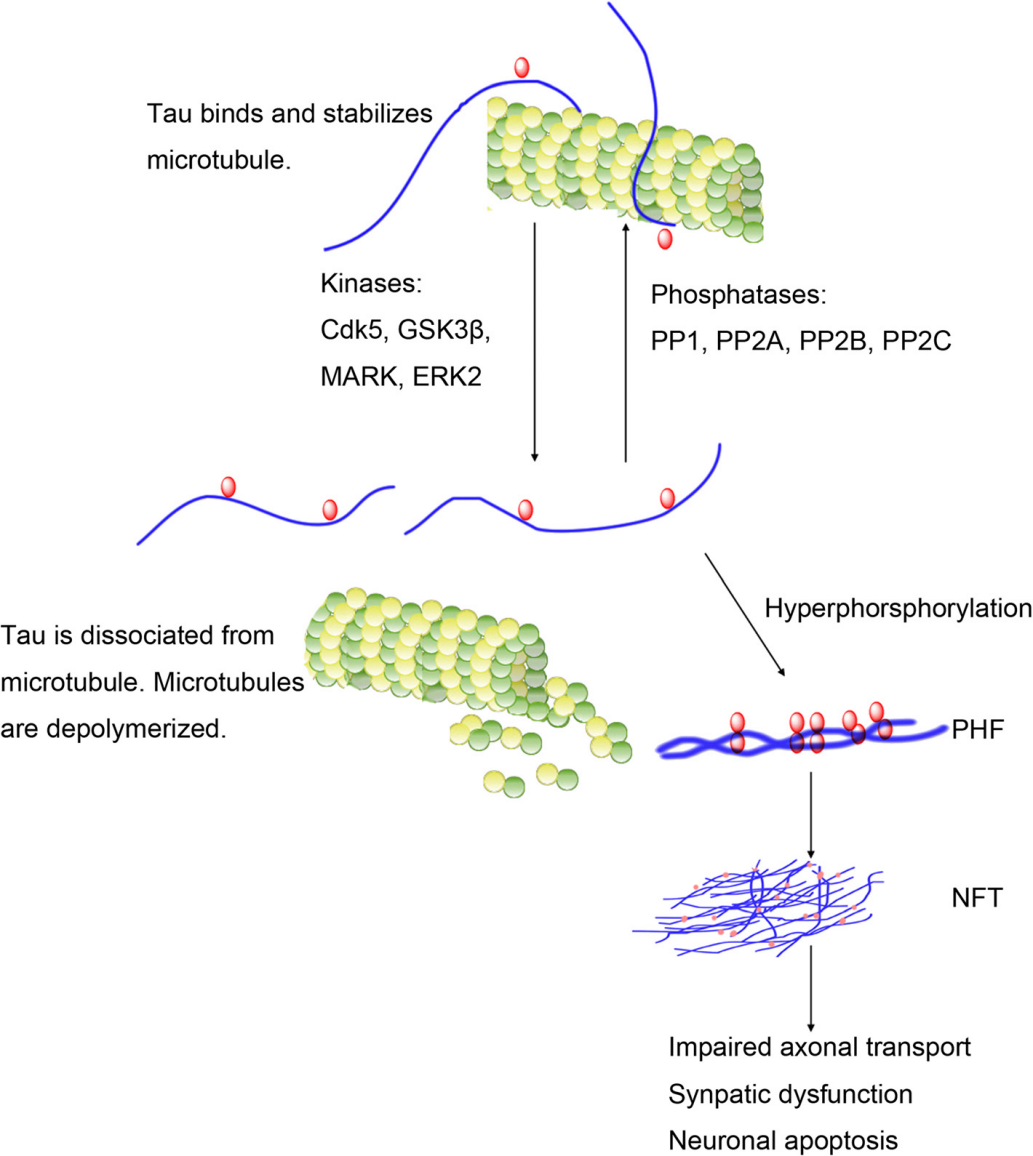
**Tau-mediated neurodegeneration. **Physiologically tau protein can bind and thereby stabilize microtubules (MTs). The attachment of tau to MT is regulated by its phosphorylation level. Phosphorylation of tau mediated by kinase (Cdk5, GSK3β, MARK and ERK2) may lead to the detachment of tau from MT and hereby cause MT depolymerization. Conversely, phosphatase (PP1, PP2A, PP2B and PP2C) will reduce the phosphorylation level of tau and restore the binding ability of tau for MT. Such equilibrium between the roles of kinases and phosphatases is disrupted under pathological condition, and increase in the kinase activity and decrease in the phosphatase activity will cause tau hyperphosphoryation. Hyperphosphorylated tau protein is misfolded and forms β-sheet-containing structure paired helical filaments (PHFs). These structure transitions will lead to more organized aggregates, and eventually develop neurofibrillary tangles (NFT) inside neurons. NFT will impair normal axonal transport, disrupt synaptic plasticity, and finally induce cell loss.

In parallel with senile plaques and cerebral amyloid angiopathy, the hyperphosphorylation of tau protein and formation of intraneuronal neurofibrillary tangles (NFTs) represents another characteristic neuropathological feature in AD brain (see Figure 1). Tau is a microtubule-associated protein (MAP). Aberrantly phosphorylated tau is the main constituent of the aggregated paired helical filaments (PHF) that comprises NFT. Despite decades of intense research that strongly implicates NFT in underlying pathogenesis of AD and other neurodegenerative diseases (so called tauopathies) [1], there has been controversy as to whether NFTs or Aβ plaques are the primary cause of AD, and the interrelationship between these two pathologies remains largely elusive [2].We have re-evaluated the role of Aβ in AD in our previous review [3]. In this review, we further discussed the recent advances in tau-mediated AD pathology with focusing on the propagation of tau pathology, tau hyperphosphorylation and the relationship between tau and Aβ.

## Physiological functions of tau

MAP tau localizes predominantly in the axons of neurons with the primary function in maintaining microtubules (MTs) stability. It is necessary for neurite outgrowth. Tau presents six main isoforms in the human brain (ranging between 352 and 441 amino acid residues), according to the alternative splicing, and differs by having 3 or 4 semi-conserved repeats of 31 residues in the MT-binding assembly domain and 0-2 insertions in the N-terminal projection domain [4,5]. Between the projection domain and the microtubule-binding domain lies a basic proline-rich region. MT-binding domain is important for promoting microtubule assembly, although it binds to microtubules only with low affinity. The various isoforms appear differentially during development. The ratio of 3R and 4R tau isoforms is 1:1 in most regions of the adult brain, and deviations from this ratio are characteristic of neurodegenerative tauopathies [6].

The MT-binding ability of tau is Post-translationally regulated primarily by serine/threonine-directed phosphorylation, which can effectively modulate the binding affinity of tau for MTs [7], although other Post-translational modifications, such as glycosylation, may also have a direct impact on the dynamic equilibrium of tau on and off the MTs. Dynamic tau phosphorylation occurs during brain embryonic development [8]. It is substantially increased during the development of the fetal brain while decreased gradually in the adult brain [9]. Furthermore, tau has profound effects on axonal transport, function and viability of neurons and their highly extended processes [10]. Tau is key to the sophisticated transport machinery that allows signaling molecules, trophic factors and other essential cellular constituents, including organelles (for example mitochondria and vesicles), to travel along the axons. Tau also interacts with various other proteins in addition to tubulin, including the SH3 domains of Src family tyrosine kinases [11,12].These results strongly suggest that tau has a potential role in cell signaling. In addition, recent studies also demonstrated that tau plays functional roles in nucleolar organization and chromosome stability [13,14].

## Pathological aggregation of tau

### Free tau, PHF and NFTs

When the balance between tau phosphorylation and dephosphorylation is changed in favor of the former, tau is hyperphosphorylated and the level of the free tau fraction is elevated. Tau has been found to be phosphorylated at over 30 serine/threonine residues and approximately half of these are canonical sites for proline-directed protein kinases(PDPKs), suggesting important roles for PDPKs and protein phosphatases in the abnormal hyperphosphorylation of tau [15]. Hyperphosphorylated tau aggregates into NFTs through pretangles (nonfibrillary tau deposits) and PHF [16].

### Phosphorylation and dephosphorylation of tau

Various other pathological events, including Aβ-mediated toxicity, as well as oxidative stress and inflammation, may be able to trigger or contribute (independently or in combination) to an abnormal detachment of tau from the MTs [17-20]. For example, it has been suggested that oxidative stress could be responsible for detrimental covalent modifications of tau, which include the formation of intermolecular disulphide bridges and tyrosine nitration. Such modifications are likely to cause misfolding, hyperphosphorylation and aggregation, and thereby contribute to abnormal disengagement of tau from MTs, as well as to the formation of aggregates. Although oxidative stress is often regarded as an upstream event relative to tau pathology, recent studies have revealed that pathological tau may interfere with mitochondrial function and induce oxidative stress [21-23]. In addition, lifetime stress, endoplasmic reticulum stress and hypersecrete glucocorticoids exposure also influence tau hyperphosphorylation [24,25].

#### Kinases of tau

Several lines of in vitro data have shown that many kinases are involved in phosphorylation of tau, though it is not yet clear if it is also physiologically or pathologically true in vivo. Nevertheless, cyclin-dependent kinase 5 (CDK5), glycogen synthase kinase 3 (GSK3), the microtubule-affinity-regulating kinase (MARK) and extracellular signal-regulated kinase 2 (ERK2) have received particular attention as potential targets for disease-modifying therapies using inhibitory compounds [7,26].

P35 and p39 proteins are expressed almost exclusively in postmitotic neurons and have been identified as CDK5 activators [27]. Elevated cellular calcium levels trigger the calpain-mediated cleavages of p35 and p39 to form the more stable p25 and p29 fragments [28,29]. Indeed, calpain activation, p25 accumulation and elevated CDK5 activity have all been observed directly in the AD brain [30,31]. This has also been evident in the transgenic mice that overexpress human p25 that exhibit increased CDK5 activity, hyperphosphorylation of tau, neurofilament and cytoskeletal disturbances [32]. Inducible transgenic mice overexpressing p25 in the postnatal forebrain also exhibit neuronal loss and caspase-3 activation, accompanied by hyperphosphorylation of endogenous tau, accumulation of aggregated tau, and the progressively developed neurofibrillary pathology [33]. Together, these data suggest CDK5-p25 pathway is a crucial component of AD pathophysiology. Interestingly, mice overexpressing p35 as well as tau and CDK5 do not show increased tau phosphorylation, and the cdk5/p35 could not cause neurodegeneration in mouse brain, suggesting that cdk5/p35 might not be a major protein tau kinase [34].

Cdk5 modulates tau hyperphosphorylation via the inhibitory regulation of GSK3 [35]. GSK3 has two isoforms, GSK3α and GSK3β. In transfected mammalian cells, GSK3α and GSK3β could contribute to the formation of PHF [36,37]. Transgenic mice with elevated GSK3β expressions show increased tau phosphorylation and deficits in spatial learning [38]. In newborn AD transgenic mouse models, knockdown of GSK3α and GSK3β reduces tau phosphorylation and tau misfolding, while the knockdown of GSK3α, but not GSK3β, leads to reduced senile plaques formation. These data demonstrate that GSK3β only modulates NFT formation, while GSK3α contributes to both senile plaques and NFT pathogenesis [39].

MARK phosphorylates tau on non-Ser/Thr-Pro sites and plays a crucial role in regulating tau’s function. MARK selectively phosphorylates a KXGS motif, which is presented in each MT-binding domain of tau. Overexpression of MARK promotes tau phosphorylation at KXGS motifs and disrupts the microtubule array in vivo [40]. Although little is known so far about the upstream events that act through MARK to regulate tau phosphorylation, one recent study demonstrated that GSK3β is substantially responsible for phosphorylating Ser-262 of tau through activation of MARK2 [41].

ERK2 is highly expressed in neurons and plays an important role in regulating tau functions and tau phosphorylation. This kinase can promotes tau phosphorylation and hereby reduce the ability of tau in stabilizing microtubules [7].

Besides the roles either directly or indirectly in modulating tau phosphorylation, recent studies have revealed that the kinases mentioned above are also associated with APP cleavage. For instance, GSK3, especially GSK3α, involves in APP processing, and the production of Aβ peptides can be significantly reduced by interfering APP cleavage at the gamma-secretase step with lithium, a GSK3 inhibitor [42]. Inhibition of GSK3α may thus offer a new approach to reduce the formation of both amyloid plaques and NFTs. CDK5-p25 can also modulate the production of Aβ by increasing APP phosphorylation at Thr668 [43].

#### Phosphatases of tau

It has been identified that a number of phosphatases, such as protein phosphatase (PP) 1, PP2A, PP2B and PP2C, could potentially drive the reverse and dephosphorylation of tau. Their activities were found to be decreased about 20-30% in AD brain [44].

PP2A is co-localized with tau and microtubules in the brain and is apparently the most active enzyme in dephosphorylation of tau. In AD brain, both the expression and activity of PP2A are decreased. Tau can be abnormally hyperphosphorylated if I1PP2A, a 249-amino acid long endogenous inhibitor of PP2A**,** is increased [45]. One recent study reported that PP2A could be inactivated via phosphorylation of its catalytic subunit at Y307. This PP2A inactivation can be mediated by Aβ deposition or estrogen deficiency in the AD brain. Moreover, the inactivation of PP2A consequently compromise dephosphorylation of abnormally hyperphosphorylated tau, therefore lead to neurofibrillary tangle formation [46].

## Other Post-translational modification of tau

In addition to tau phosphorylation, different types of post-translational modifications including acetylation, glycosylation, glycation, prolyl-isomerization, cleavage or truncation, nitration, polyamination, ubiquitination, sumoylation, oxidation and aggregation can regulation the function of tau [47-49]. Of these modification, tau acetylation is of great importance for tauopathy. Tau acetylation has recently been found to prevent p-tau from degradation and modulate the activities of kinase, implicating a central role in tauopathy [50].

### The toxicity of tau

NFTs are considered to be responsible for the toxic effects of tau in AD for a long time, but recent findings suggest that this might not be all the fact. Santacruz et al. by using a strain of mice bearing a mutant human tau gene combined with regulatory sequences that allowed it to be turned off by the antibiotic doxycycline, demonstrated that the animals’ memories are improved and the neuronal losses are halted when the mutant tau gene is switched off, but with no effect on NFT accumulation [51]. One study also showed that inhibition of tau phosphorylation is able to prevent the typical motor deficits and markedly reduce soluble aggregated hyperphosphorylated tau in the tau transgenic mice [52], suggesting that PHF or other soluble lower-mass hyperphosphorylated tau aggregates are neurotoxic. Meanwhile, increasing evidence has revealed that tau-mediated neurodegeneration may result from the combination of gain-of-toxic function acquired by the aggregates or their precursors and the detrimental effects that arise from the loss of the normal function(s) of tau in the disease state [53,54].

## Propagation of tau pathology

NFTs have a hierarchical pattern of accumulation in vulnerable neurons. The neurons in layer II of the entorhinal cortex (EC-II) are considered as the first to be affected. Later, the lesions appear to spread to limbic and association cortices [55]. However, the exact mechanism involved in the pattern of propagation is incompletely understood. Recently, several studies have showed that the intracellular protein aggregates of tau can spread by a prion-like mechanism in the brain. The extracellular tau aggregates can enter cells through endocytosis and trigger the misfolding and aggregation of intracellular tau in cell culture experiments [56-58]. In another study [59], de Calignon et al. generated a transgenic mouse model in which overexpression of human mutant tau (P301L) is restricted to EC-II (named rTgTauEC mouse) to investigate the disease progression. They found that tau pathology progresses from the EC neurons expressing the human transgene to the nearby neurons, and then to neurons located downstream in the synaptic circuit, for instance the dentate gyrus, hippocampus, and cingulate cortex [59]. These findings provide useful information for understanding the hierarchical patterns of tau-mediated neurodegeneration in AD.

## Tau and Aβ

Both senile plaques and NFTs are predominant pathologic characteristics of AD. Connections between Aβ toxicity and tau pathology have repeatedly been proposed. However, the underlying mechanisms have not yet been fully established, and this remains one of the most challenging conundrums of AD research.

It is increasingly recognized that reduction in tau levels can alleviate memory loss in the AD mouse model. Mucke and his colleagues found that decreasing endogenous tau prevents behavioral deficits in transgenic mice with mutant APP, without altering their high Aβ levels. Tau reduction also protected both APP transgenic AD model and nontransgenic mice against excitotoxicity. The absence of tau somehow prevents the behavioral deficits that would otherwise occur in animals [60]. New lines of investigation support the notion that tau malfunction, in addition to being independently capable of producing neurodegeneration even in the absence of Aβ deposits or other pathological events, could be a key mediator of neurodegeneration in response to other upstream events, including Aβ-induced neurotoxicity [20].It has been shown that Aβ can bind to tau and form a stable complex both in vitro and in AD brain [61]. The complex enhances tau phosphorylation via GSK-3β signaling, suggesting that Aβ lies in the upstream of tau pathology. This has been further supported by the study of Bolmont et al. in which intracerebral injection of brain extracts from APP transgenic mice induced the formation of NFT in mutant tau transgenic mice [62]. The mutant tau and APP double transgenic mice exhibit more significant increasing in tau pathology than the mutant tau transgenic mice predominantly in the area with high amyloid burden, while the double transgene do not lead to up-regulation of amyloid load as compared with the mutant APP transgenic mice. Aβ and phosphorylated-tau have been observed to be co-localized in synaptic terminals of AD brains [63]. Aβ can result in the transcriptional up-regulation of a gene named dual-specificity tyrosine-regulated kinase 1A (DYRK1A), further leading to tau phosphorylation [64]. Moreover, microtubule disassembly, one of pathological functions of tau, is initiated by prefibrillar Aβ [18]. Also, tau is required for the cytotoxicity of hybrid oligomers formed by Aβ_3(pE)–42_ and Aβ_1–42_[65]. Recent studies showed that tau deficiency in *tau*^*-/-*^ mice and truncated tau in transgenic mice both lead to disruption of postsynaptic targeting of Fyn kinase and attenuation of Aβ toxicity, indicating that tau is a mediator of Aβ toxicity [66].

All these suggest that Aβ may drive tau pathology, and tau can mediate Aβ toxicity, implicating a cooperation between Aβ and tau for AD pathology. In this regard, it has been demonstrated that Aβ and tau could synergistically impair mitochondrial respiration in a triple transgenic Alzheimer's disease mice [67,68]. However, the exact mechanisms underlying such an interaction of Aβ and tau need further investigation.

## Conclusion

As a most common neurodegenerative disorder, AD characterized by hyperphosphorylation of tau protein and formation of NFTs are collectively termed “tauopathy”. This review highlights the recent advances in tau-mediated AD pathology, including tau hyperphosphorylation, propagation of tau pathologyand the relationship between tau and Aβ. Tau plays an unequivocal role in AD, but the mechanisms of tau that induce dysfunction and death of neurons remain incompletely understood. Future researches can focus on the precise mechanisms of tau involved in the disease pathogenesis, which may eventually lead to the development of new therapeutic strategies for tauopathies of AD.

## Competing interests

The authors declare that they have no competing interests.

## Authors’ contributions

YLD, SZD and FG collected the reference materials and drafted the manuscript. YHH and ZZ conceived of the study, and participated in its design and coordination and helped to draft the manuscript. All authors read and approved the final manuscript.
